# Questionnaire survey about use of an online appointment booking system in one large tertiary public hospital outpatient service center in China

**DOI:** 10.1186/1472-6947-14-49

**Published:** 2014-06-09

**Authors:** MinMin Zhang, CongXin Zhang, QinWen Sun, QuanCai Cai, Hua Yang, YinJuan Zhang

**Affiliations:** 1 Gastroenterology Department of Shanghai Changhai Hospital, Second Military Medical University, 168 Changhai Road, Shanghai 200438, China; 2 Outpatient Service Center of Shanghai Changhai Hospital, Second Military Medical University, Shanghai, China; 3 Department of Physics and Mathematics, College of Basic Medical Science, Second Military Medical University, Shanghai, China; 4 Clinical Epidemiology and Evidence-based Medicine Center, Second Military Medical University, Shanghai, China

**Keywords:** outpatient care, Waiting list, Online appointment booking system, Questionnaire, Healthcare reform

## Abstract

**Background:**

As a part of nationwide healthcare reforms, the Chinese government launched web-based appointment systems (WAS) to provide a solution to problems around outpatient appointments and services. These have been in place in all Chinese public tertiary hospitals since 2009.

**Methods:**

Questionnaires were collected from both patients and doctors in one large tertiary public hospital in Shanghai, China.Data were analyzed to measure their satisfaction and views about the WAS.

**Results:**

The 1000 outpatients randomly selected for the survey were least satisfied about the waiting time to see a doctor. Even though the WAS provided a much more convenient booking method, only 17% of patients used it. Of the 197 doctors surveyed, over 90% thought it was necessary to provide alternative forms of appointment booking systems for outpatients. However, about 80% of those doctors who were not associated professors would like to provide an ‘on-the-spot’ appointment option, which would lead to longer waits for patients.

**Conclusions:**

Patients were least satisfied about the waiting times. To effectively reduce appointment-waiting times is therefore an urgent issue. Despite the benefits of using the WAS, most patients still registered via the usual method of queuing, suggesting that hospitals and health service providers should promote and encourage the use of the WAS. Furthermore, Chinese health providers need to help doctors to take others’ opinions or feedback into consideration when treating patients to minimize the gap between patients’ and doctors’ opinions. These findings may provide useful information for both practitioners and regulators, and improve recognition of this efficient and useful booking system, which may have far-reaching and positive implications for China’s ongoing reforms.

## Background

The lack of effective referral systems and the uneven distribution of medical investment, resources and talent between rural and urban areas in China cause patients to crowd into larger hospitals. This has made the queue time for an outpatient appointment one of the biggest causes for complaint among the Chinese public. Nearly all of the country's highest regarded doctors and most advanced facilities are located in urban areas, causing patients to scramble for limited medical resources. Many patients are even forced to queue late at night. If they can afford it, some patients will pay others to wait in their place. Some even develop personal relationships with hospital staff in the hope of getting preferential treatment. These issues have caused the relationship between patients and doctors to deteriorate [1]. There have even been incidents of knife attacks at hospitals in China, as people express their anger over the inadequacies of China's medical system [2]. “Too difficult to see a doctor” has become one of the top issues in China’s opinion polls [3,4].

In recent years, China has been in the process of implementing reforms to the healthcare system [5]. The aim of the reforms was to provide basic and convenient medical care, including easy access to a doctor. One of the proposals, posted on the Chinese Ministry of Health website on 2009, says that all the Chinese mainland's top state-owned hospitals should establish their own outpatient appointment-booking service and gradually broaden the service to include specialists. With the rapid development of the internet over the last few years, some hospitals tested the use of web-based appointment systems (WAS) for outpatients [6], and all public tertiary hospitals, supported by the Ministry of Health, began to use WAS in 2009, hoping to alleviate the problem of long outpatient waiting times.

Compared with other methods for booking appointments, WAS provided by hospital, usually displaying the largest variety of information on a hospital’s website, such as disease categories and physician treatments, should be much more sufficient [7,8]. However, only a small number of outpatient appointments were actually made through this system. Even though knowledge of the system was widespread, most patients still chose walk-in registration, which makes WAS difficult to implement and limited in its effect [9].

An important step in improving the responsiveness of hospitals to patients’ needs is to ask the patients themselves about their experiences and opinions. In addition, many physicians have opinions about the booking systems, which are not always considered, but in fact have huge impact on the way the policy is implemented. This pilot study therefore aimed to measure outpatients’ satisfaction and views about the WAS, using questionnaires collected from both outpatients and doctors from one large tertiary public hospital. We also wanted to find ideas for future reforms of the Chinese health system that would improve patients’ overall satisfaction with medical services and improve trust.

## Methods

Using a structured questionnaire, we evaluated attitudes and experiences among outpatients at the Changhai Hospital in relation to WAS and the conventional walk-in process. The Changhai Hospital is a large tertiary public hospital located in Shanghai. The facility's outpatient center includes areas for registration, units for clinical consultation, medical examinations and imaging. It has an average of 10,000 daily outpatient visits including patients with Shanghai Cities and Towns’ medical insurance as well as patients who pay for themselves. The study was authorized and approved by the Ethics Committee of Changhai Hospital and carried out from October 2011 to January 2012.

### Study population

Based on the average number of outpatient visits, and the number of outpatient doctors, the sample size was set at 1000 patients and 200 doctors according to the thought of target response precision. Considering the influence of different specialties as a hierarchical factor, the sample number of doctors from different specialties was in proportion to the numbers working in different outpatient specialties throughout the hospital.

### Study design

We instructed trained research assistantsat each consulting unit in the outpatient service center to interview the current patients every 45 minutes to complete the questionnaire.If the patient is an old person, the assistants will help him/her to complete the questionnairewith his/her answer. The questionnaire, called the ‘Changhai Outpatient Experience Questionnaire’ (CHOEQ) (Additional file 1), was a 42-item previously validated questionnaire focusing on feedback from patients on what they experienced in the course of receiving outpatient care, derived from the Patient Satisfaction Questionnaire. This is a 55-item instrument that assesses patients’ attitudes toward physicians and medical care services [10]. The physicians who provided outpatient service during the same period were asked to complete the ‘Changhai Doctor Experience Questionnaire’ (CHDEQ) (Additional file 2), which contained 24 items and focused on their views about outpatient appointments. All questionnaires were administered anonymously to discourage an acquiescent or socially-desirable response bias [11].

The CHOEQ measured the main specific aspects of outpatient hospital experience: expected and actual waiting and consultation time, satisfaction with behavior of staff and, in particular doctors, preferred appointment booking system and suggestions about WAS, and an overall rating for the visit.The booking system can be divided into four main categories. Mode 1: On-the-spot appointment mode (registration desk); Mode 2: On-the-spot appointment mode (consulting room); Mode 3: WAS; Mode 4: Self-help booking on machine in outpatient department.About WAS, patients can select which consultant they prefer, with only a rough estimate of appointment time, or a particular appointment time. In addition, patient demographic information (age, gender, marital status, educational level) and other information, such as socioeconomic status, and payment mode of medical fee were included. The CHDEQ mainly focused on satisfaction with the general systems and layout used in the outpatient department, opinions about the treatment processes and assistance there, what they consider to be a reasonable consultation time per patient, and views about different appointment booking systems.

### Analysis of data

SAS 9.2 was performed to process and analyze research data in this study. Measurement data were shown as mean ± standard deviation, median, and minimum–maximum.Depending on the normality and homogeneity of variance of the data, the t test, revised t test, or Wilcoxon rank-sum test were used for comparisons between two groups of independent samples, with variance analysis or the Wilcoxon rank-sum test used for larger groups. Counting categorical data were shown as a frequency (%). The chi-square test, revised chi-square test, or Fisher’s exact probability test were used for comparisons between groups. Ranked data were shown as frequency (%), and we applied the Mann–Whitney Wilcoxon rank-sum test and Kruskal-Wallis statistics testfor comparisons between two groups and larger groups, respectively. Comprehensive satisfaction values were calculated by rank analysis for ordinal data.It needs to be noticed that the satisfaction valueis an inverse scale. The higher the score the lower the satisfaction. A P-value < 0.05 was considered statistically significant.

## Results

Responses were obtained from 958 (95.8%) of the 1000 randomly chosen participants. Of the 200 doctors, 197 completed CHDEQ, a response rate of 98.5%. Some of the questionnaire data had input errors or was missing, with an input error ratio of about 1% of the patients, 57.8% (n = 517) were female, 64% (n = 642) were from households with a monthly income of less than 5000 RMB Yuan (US$793.7) per capita, and 54% (n = 479) had senior high school as their highest level of education. The percentage of patients who pay by out-of-pocket was 38% (n = 346). Half were from local, and over 70% had visited the outpatient department before (Table 1). Of the doctors, 60.8% were male, and over half were either an attending physician or resident (Table 1).

**Table 1:** Characteristics of study population

**Variable**	**Number**	**%**
Patients (n = 958)		
Gender (n = 895)		
Male	378	42.23
Female	517	57.77
Age (year, n = 926)		
<18	38	4.10
18-25	143	15.44
26-35	237	25.59
36-45	163	17.60
46-55	155	16.74
56-65	118	12.74
>65	72	7.78
Marriage (n = 932)		
Single	191	20.49
Married	577	61.91
Divorced/Separated	139	14.91
Widowed	25	2.68
Education level (n = 887)		
Post graduated or above	60	6.76
Undergraduate or Junior College	348	39.23
Technical secondary school or senior high school	226	25.48
Junior high school	175	19.73
Primary school or below	78	8.79
Place of residence (n = 921)		
Local	475	51.57
Not local, immigrated from other cities	193	20.96
Not local, just visit Shanghai	253	27.47
Way of paying (n = 899)		
Out-of-pocket	346	38.49
Medical insurance	513	57.06
Others payment	40	4.45
Monthly income (Yuan, n = 886)		
< 1000	78	8.80
1001-3000	307	34.65
3001-5000	257	29.01
5001-10000	172	19.41
> 10000	72	8.13
Is this the first time visiting? (n = 898)		
Yes	257	28.62
No	641	71.38
Doctors (n = 200)		
Professional title (n = 197)		
Attending doctor or Resident	114	57.87
Associate Professor	45	22.84
Professor	38	19.29
Gender (n = 194)		
Male	118	60.82
Female	76	39.18
Specialty (n = 194)		
Surgery	61	31.44
Internal medicine	59	30.41
Gynecology and obstetrics Department, Otolaryngology Department, Stomatology Department, Craniocerebral surgery Department, and Burns surgery Department	32	16.49
Neurology Department and Epidemiology Department	13	6.70
Imageology Department and Experimental Diagnosis Department	7	3.61
Other Departments	22	11.34

### Levels of satisfaction about the services available

**Figure 1:**
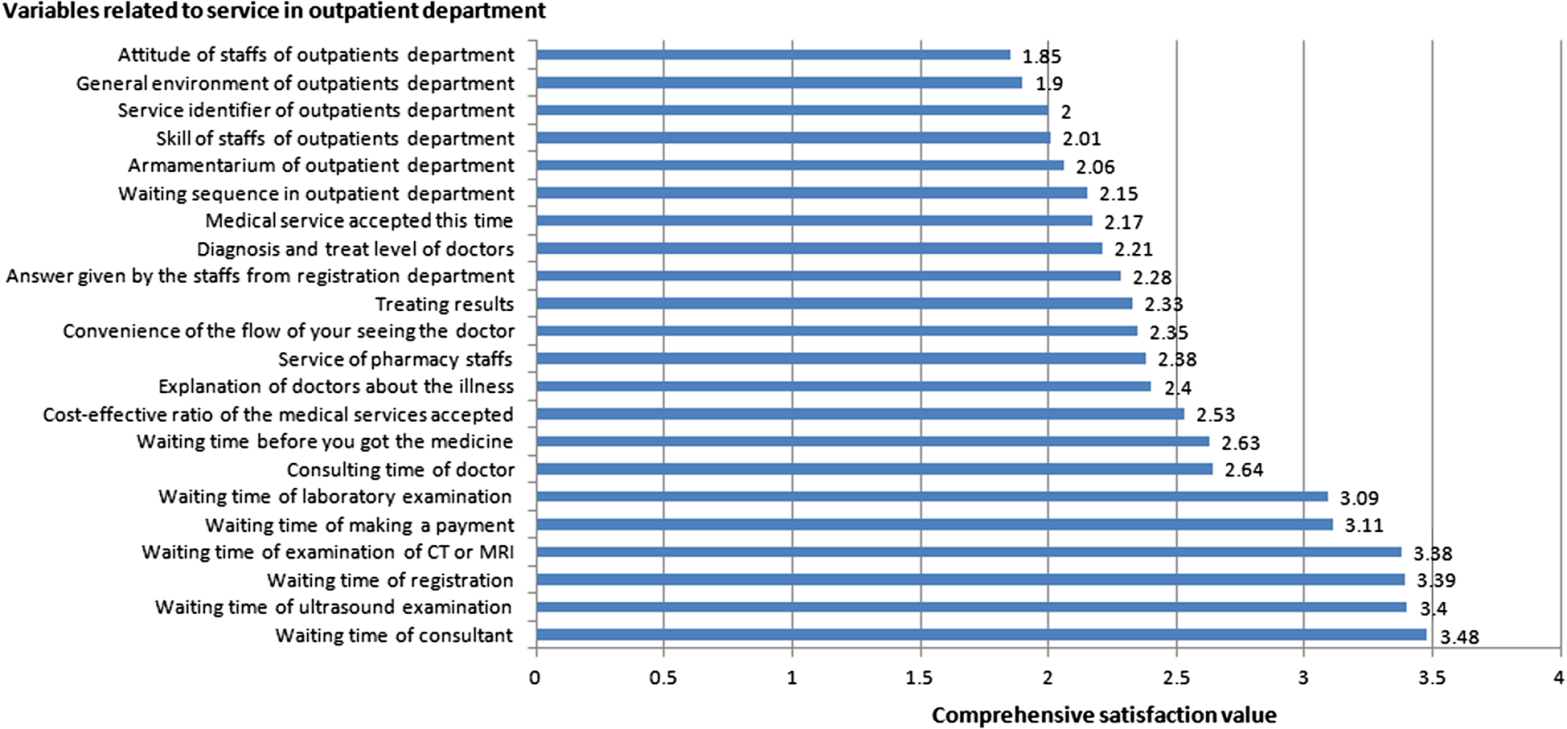
**Ranking of satisfaction of outpatients about the services in the outpatient department.** A comprehensive relative satisfaction value was calculated through the ordered variable rank analysis method. The higher the comprehensive satisfaction value was, the lower the real satisfaction.

**Figure 2:**
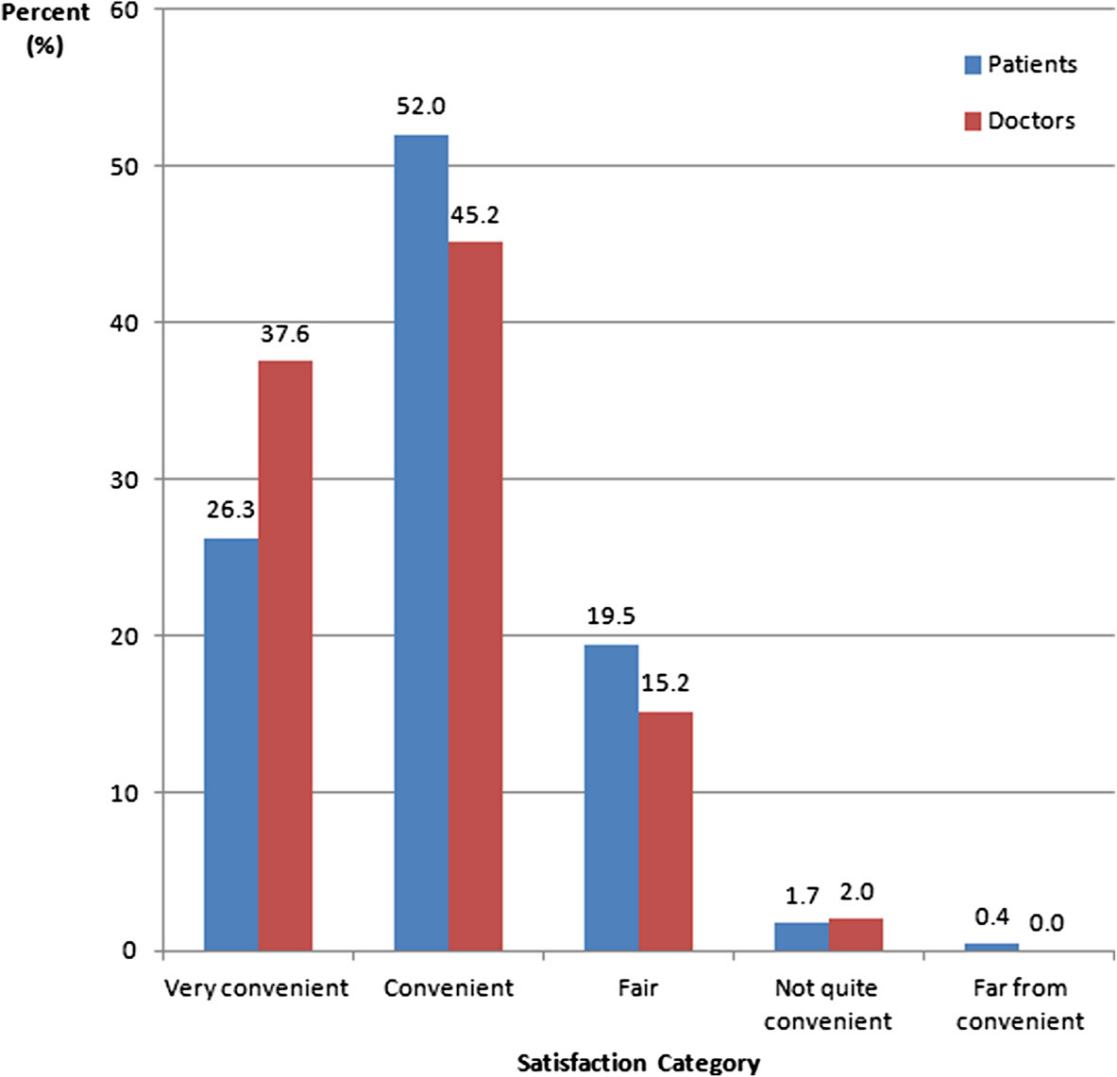
Comparison of satisfaction with current medical services shown by patients and doctors.

Even though services were considered generally ‘satisfactory’, the levels of satisfaction were lowest about how long patients waited to see a doctor. As laboratory tests and ultrasound examinations are faster diagnostic tests than other types of procedures/exams in China, most patients choose to have these tests immediately after their consultation. Further investigation revealed that the six items with the lowest levels of satisfaction were all about waiting times, and included the time spent waiting for a laboratory test, to pay, for a CT or MRI scan, to register to see a doctor and for ultrasound examinations (Figure 1). Patients could tolerate waiting to see a doctor for no more than 30 minutes. There were no significant differences in views by patients’ age and occupation. However, patients who were not local, but lived permanently in Shanghai tolerated a slightly lower waiting time to see the doctor than those who were immediately local or not from Shanghai (P = 0.012). Patient satisfaction was significantly lower than that of doctors about the services available (P = 0.004) (Figure 2).

### Use and opinions of the WAS

Of 958 patients, only 35% had used any of the available systems to book an appointment (n = 310). Of those, over 70% preferred the walk-in mode (n = 217). Only 17% patients preferred WAS (n = 53). There was no significant difference in terms of marital status, occupation, way of paying and family income, although education level did seem to affect choice of booking system (Table 2). Patients had preferences for different ways of getting appointments depending on their opinion about the WAS. Patients under 18 years old or over 65 years old tended to use a mode by which they could select which doctor to see. Patients in other age groups tended to choose a mode which auto-selected precise time slots for appointments. Education level had influence on choice: patients whose highest level of education was grade school preferred to use a system that automatically selected which doctor they would see (P < 0.001). Others tended to choose a method that automatically selected a time slot (Table 3).

**Table 2:** Difference between systems used to book appointments by patients’ demographic factors

**Variables**	Mode 1* n (%)	Mode 2* n (%)	Mode 3* n (%)	Mode 4* n (%)	**P Value**
**Age (year)**					0.367
<18	2(1.26)	2(3.77)	0(0.00)	1(5.56)	
18-25	22(13.84)	2(3.77)	7(17.50)	2(11.11)	
26-35	50(31.45)	12(22.64)	13(32.50)	4(22.22)	
36-45	27(16.98)	7(13.21)	6(15.00)	5(27.78)	
46-55	26(16.35)	10(18.87)	6(15.00)	3(16.67)	
56-65	20(12.58)	15(28.30)	7(17.50)	2(11.11)	
>65	12(7.55)	5(9.43)	1(2.50)	1(5.56)	
**Marriage**					0.341
Single	29(18.35)	3(5.66)	6(15.38)	2(11.11)	
Married	101(63.92)	38(71.70)	28(71.79)	12(66.67)	
Divorced / Separated	23(14.56)	12(22.64)	4(10.26)	4(22.22)	
Widowed	5(3.16)	0(0.00)	1(2.56)	0(0.00)	
**Education level**					0.009
Post graduated or above	15(9.87)	0(0.00)	7(17.50)	1(6.25)	
Undergraduate or Junior College	56(36.84)	20(40.00)	17(42.50)	2(12.50)	
Technical secondary school or senior high school	42(27.63)	15(30.00)	8(20.00)	3(18.75)	
Junior high school	32(21.05)	9(18.00)	7(17.50)	6(37.50)	
Primary school or below	7(4.61)	6(12.00)	1(2.50)	4(25.00)	
**Place of residence**					0.607
Local	87(55.41)	34(64.15)	20(50.00)	9(52.94)	
Not local, immigrated from other cities	33(21.02)	6(11.32)	10(25.00)	5(29.41)	
Not local, just visit Shanghai	37(23.57)	13(24.53)	10(25.00)	3(17.65)	
**Way of paying**					0.374
Out-of-pocket	53(34.87)	12(22.64)	15(39.47)	8(44.44)	
Medical insurance	94(61.84)	37(69.81)	21(55.26)	10(55.56)	
Others payment	5(3.29)	4(7.55)	2(5.26)	0(0.00)	
**Monthly income per capita (Yuan)**					0.101
< 1000	11(7.33)	4(8.00)	4(10.26)	0(0.00)	
1001-3000	49(32.67)	20(40.00)	8(20.51)	8(47.06)	
3001-5000	49(32.67)	11(22.00)	9(23.08)	3(17.65)	
5001-10000	28(18.67)	9(18.00)	12(30.77)	1(5.88)	
> 10000	13(8.67)	6(12.00)	6(15.38)	5(29.41)	

*Mode 1: On-the-spot appointment mode (registration desk); Mode 2: On-the-spot appointment mode (consulting room); Mode 3: WAS; Mode 4: Self-help booking on machine in outpatient department.

**Table 3:** Preference for different registration modes using online booking

**Variables**	**Auto selecting different serial number mode**		**Auto selecting different consultant time interval mode**		**P Value**
	**n**	**%**	**n**	**%**	
**Age (years)**					0.036
<18	14	5.41	9	3.00	
18-25	44	16.99	47	15.67	
26-35	60	23.17	74	24.67	
36-45	47	18.15	59	19.67	
46-55	38	14.67	56	18.67	
56-65	35	13.51	36	12.00	
>65	21	8.11	19	6.33	
**Marriage**					0.378
Single	59	22.69	63	20.86	
Married	155	59.62	192	63.58	
Divorced/Separated	41	15.77	36	11.92	
Widowed	5	1.92	11	3.64	
**Education level**					<0.001
Post graduated or above	23	9.24	16	5.48	
Undergraduate or Junior College	77	30.92	123	42.12	
Technical secondary school or senior high school	66	26.51	77	26.37	
Junior high school	52	20.88	58	19.86	
Primary school or below	31	12.45	18	6.16	
**Place of residence**					0.637
Local	130	50.19	157	52.33	
Not local, immigrated from other cities	52	20.08	62	20.67	
Not local, just visit Shanghai	77	29.73	81	27.00	
**Way of paying**					0.323
Out-of-pocket	93	37.65	110	36.91	
Medical insurance	145	58.70	171	57.38	
Others payment	9	3.64	17	5.70	
**Monthly income per capita (Yuan)**					0.311
< 1000	26	10.32	22	7.53	
1001-3000	87	34.52	104	35.62	
3001-5000	69	27.38	93	31.85	
5001-10000	44	17.46	57	19.52	
> 10000	26	10.32	16	5.48	

### Doctors’ views and opinions of the WAS

**Figure 3:**
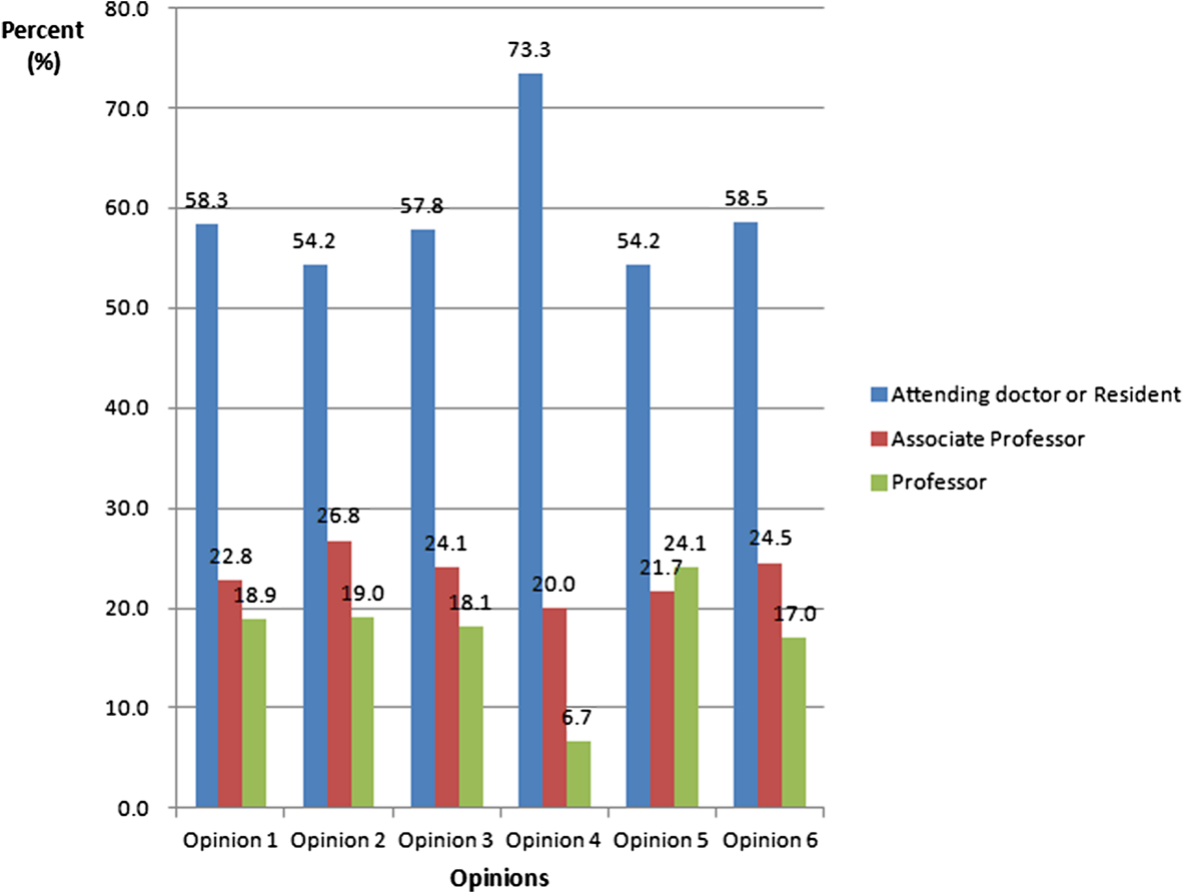
**Doctors’ opinions about the appointment booking systems and relationship with their professional title.** Opinion 1: It is necessary to provide alternative forms of appointment booking systems; Opinion 2: You will provide services on the spot for patients who visit the consulting room; Opinion 3: You used the appointment booking systems for your relatives; Opinion 4: Have heard of the appointment booking services but never used; Opinion 5: Auto-selecting different consultant orders; Opinion 6: Auto-selecting particular appointment times.

**Figure 4:**
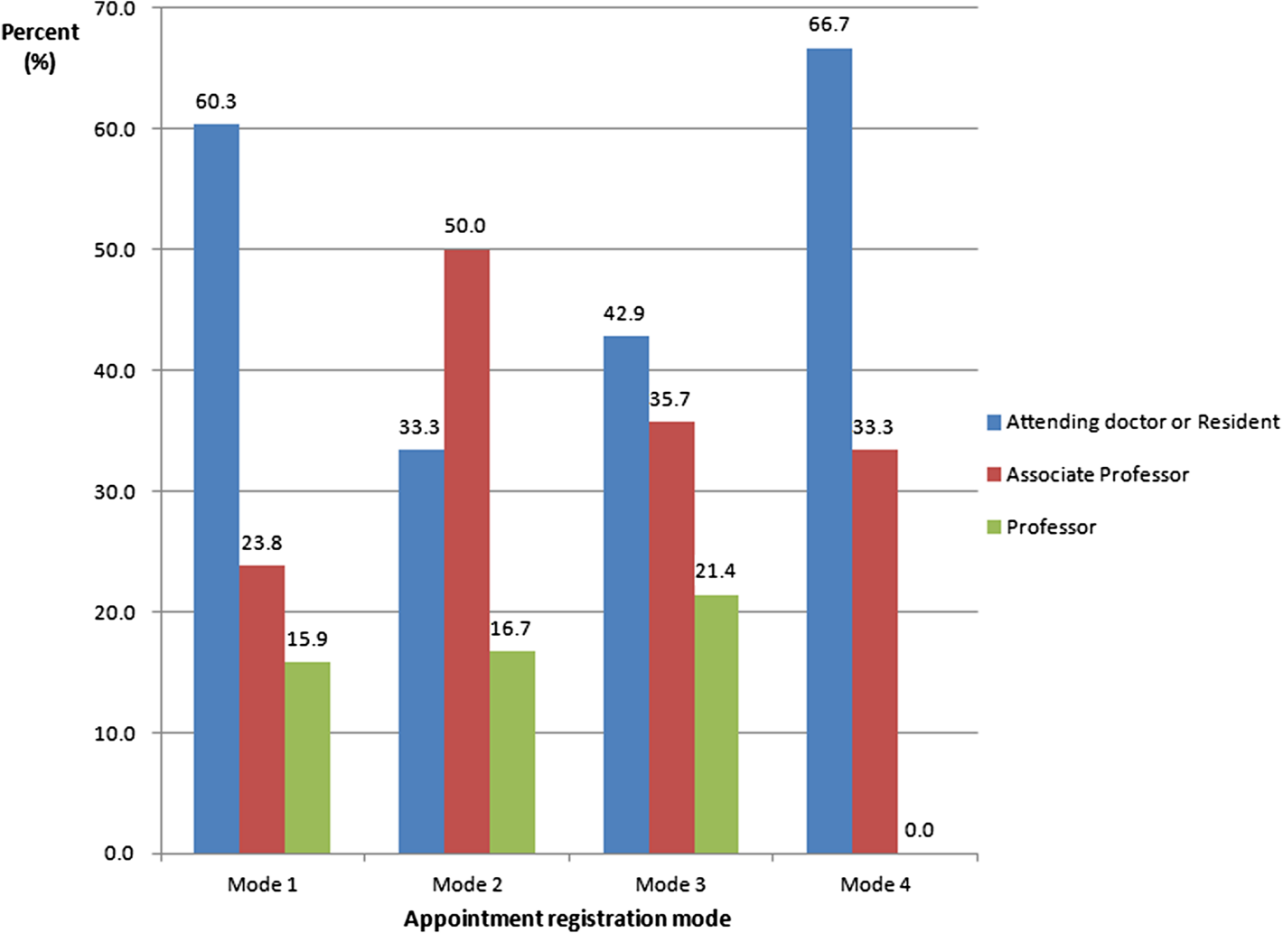
**How the use of different appointment booking systems by doctors differs by professional title.** Mode 1: On-the-spot appointment (registration desk); Mode 2: On-the-spot appointment (consulting room); Mode 3: WAS; Mode 4: Self-help booking on machine in outpatient department.

**Figure 5:**
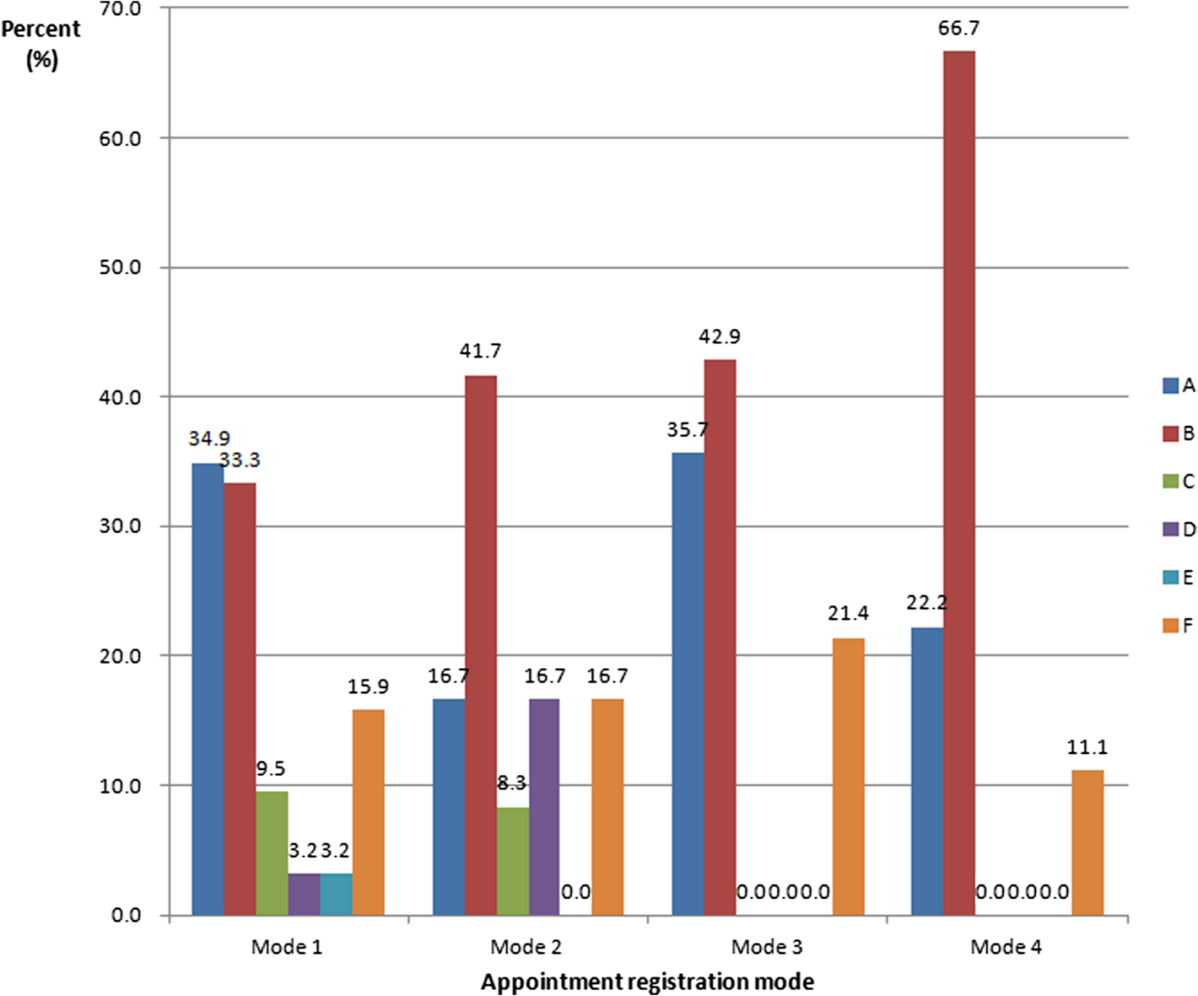
**Use of different appointment booking systems by doctors in different specialties.** A: Surgery; B: Internal medicine; C: Gynecology and obstetrics, otolaryngology, stomatology, craniocerebral surgery and burns surgery; D: Neurology and epidemiology; E: Imaging and experimental diagnosis; F: Other specialties. Mode 1: On-the-spot appointment (registration desk); Mode 2: On-the-spot appointment (consulting room); Mode 3: WAS; Mode 4: Self-help booking on machine in outpatient department.

Over 90% of doctors thought it was necessary to provide alternative ways for patients to book outpatient appointments. There was no significant difference in this between doctors with different professional titles. About 80% doctors with a title other than associate professor would prefer to provide on-the-spot appointments when patients visit their consulting room. Their views did change depending on whether they had ever used the service as a patient or patient’s relative. About 43% of doctors had used the registration services provided by Changhai Hospital Outpatients Department for their relatives, and 45% of them preferred the auto-selection of consultant or doctor (Figure 3). Eighty-nine percent of them had used the walk-in system, with most (75%) booking at the registration desk, and 14% at the consulting room. Only 17% had used the WAS (Figure 4). There were no statistically significant differences between different professional titles and different specialties (Figure 5).

## Discussion

This study is among the first to measure outpatient satisfaction and views about online and other forms of appointment booking systems, using questionnaires collected from both outpatients and doctors. The satisfaction of Chinese patients with the current state of the healthcare service they receive, and particularly around doctor-patient interaction, treatment processes, waiting times in hospital, medical facilities and hospital environment, and medical costs, is dropping [12]. According to the Chinese Ministry of Health’s “Governing Rules for the Management and Classification of Hospitals” in 1989 [13], public hospitals in China are divided into three levels. Patients have the freedom to choose the medical institutions and access higher levels of service according to need, from the community hospital up to the higher specialty hospital [14]. However, most Chinese patients in fact have no understanding of the concept of appointments and referrals, which causes problems for the tertiary public hospitals. These hospitals are supposed to provide high quality specialty medical and other healthcare services to participants in several areas, acting as high-level teaching hospitals and conduct sophisticated research. But, with demand increasing, these hospitals have had insufficient capacity to cope, resulting in serious drops in patient satisfaction [12,15]. Because outpatient services are more commonly used for effective and efficient interventions for many common health problems (especially some chronic diseases), the outpatient service center in tertiary public hospital are always overcrowded [16]. The long waiting time for a drop-in outpatient appointment in a tertiary hospital has become one of the most frequent causes for complaint among the Chinese public [17]. As patients often equate life satisfaction with their healthcare experience [15,18-20], it is not surprising that about 40% of the correspondents were not satisfied with outpatient services from the national household health survey [21].

In our research, we found that patients were least satisfied with waiting times. In other studies, patient satisfaction ratings were affected by factors such as culture, expectations, and sense of entitlement, and responses to questions on general opinion and service expectations may be affected by the actual service received [22]. However, patients always perceived the clinical staff as kinder and more compassionate if their waiting times had been shorter, and showed improved satisfaction with the care provided, even if the staff had done nothing to affect the wait [23]. In our research, we found that most patients would not tolerate waiting longer than 30 minutes, with no significant differences between patients of different ages and occupations. As waiting time is a significant component of patient satisfaction, reducing it needs to be a priority. WAS has been showed to have the ability to reduce waiting times effectively [24]. However, just as our results showed, despite the potential benefits of using the WAS, 65% of patients still chose to queue. Among patients who had used booking systems, over 70% preferred the walk-in system, with no differences depending on marital status, occupation, and way of paying and family income. One possible reason for patients not choosing the WAS was that many were unaware that they could book an appointment through the Internet. In a telephone interview survey carried out in Xijing Hospital, which is a large tertiary public hospital in Xi’an city in China, it was revealed that over half of the participants did not know that an appointment could be obtained through the Internet [24]. And patients might not have sufficient confidence in using the Internet. These findings indicate that hospitals and health service providers should make more effort to promote and encourage the use of the WAS among potential patients. Patients could be reminded about the WAS when they arrive at the registration desk in the outpatient department.

Moreover, to attract more patients to use WAS system, operation should be made even easier and more user-friendly by enhancing system service quality (for instance, webpage uploadingspeed, user-friendliness of webpages and search for information) and information variety (for example, to provide informationabout health care and outpatient pre-register systems) with Cloud Computing and APP (Application software) [8]. The current role of WAS is limited just as online appointment. If the WAS system can integrate personal health/medication records and provide patients with information related to their own health based on various curriculums (i.e. diabetes and high blood pressure) and categories (i.e. dieting and exercising) for self-control and management, disease-prevention, health remainders and checks, that would be tremendously encouraging for patients to use [8].

The other reason for patients not willing to choose WAS is the possible failure of obtaining registration for some specific day of the week or time of the day according to different registration demand. Previous study showed that most Chinese patients prefer to see a doctor on Monday or Tuesday, especially in the morning [24]. This makes it very difficult to obtain registration during these periods.How to distribute the registration demand evenly is an issue for the hospital and health providers to solve in the future. Method of computer simulation could be a good choice for medical practitioner to predict patient through put and waiting timewithout making potentially costly changes. And it has been proved to be helpful in analyzing the effects of re-organizing resources in hospital [25]. We have tried using computer simulating in outpatients’ services with Matlab software and achieved great success. And further research work is still needed.

Earlier studies showed that patients’ trust in medical services or attitude towards health policy had a significant influence on their satisfaction with waiting times in hospital [26]. However, as long waiting times have become a more general problem in China, patients’ trust in medical services and attitude towards health policy have declined, and also become a less important influence on their satisfaction with waiting times in hospital [12]. Public perceptions of fairness and trust of the health-care system erode [21]. This lack of trust may be part of the reason why patients are unwilling to use WAS. They preferred to queue for an appointment because they found this more real than the WAS, where they were just communicating with a computer. The concern that some might get preferential treatment because they knew hospital staff decreased patients’ trust still further. An increase in trust and an improvement in general attitudes towards health policy could improve patients’ attitude towards new health policies and processes, such as the WAS. The most effective way to increase patients’ trust in medical services was to improve their quality and reliability. Hospitals should therefore strengthen their management to end any unfair priority given to those who know hospital staff. The findings also suggest that more humanized hospital management might improve patients’ overall satisfaction with medical services and attitude towards health policy.

Higher physician emotional intelligence (EI) and a higher ratio of patient follow-up lead to increased patient trust [27]. Doctors provide their time and skill to treat the patient, and in return receive the patient’s compliance in the form of either further appointments or word-of-mouth recommendations to other patients. For doctors, walk-in appointments, especially those allowing appointments to be made at consulting rooms, could help them to increase patient trust. In addition, physicians’ professional competence may be developmental in nature. That could be why about 80% of doctors other than associate professors would like to provide walk-in appointments for patients who visit their consulting rooms. They consider this a sort of real-time feedback on their key competencies, such as interpersonal skills and medical expertise. The other reason that physicians may not be recommending the WAS to patients was the worry that there might be too many patients who did not attend pre-booked appointments, because it is too easy to book then not cancel. In fact, the WAS could be considered to reflect confirmation and positive feedback by patients across a wider area, not just those who can attend a walk-in clinic. It can also help to prompt doctors to contemplate or initiate changes in their daily practices. To avoid too many patients not attending, hospitals could shorten the appointment interval to reflect the percentage of patients who are forecast not to attend [28], or use an experimental computer simulation, which we have started to use in our hospital, to choose a slightly larger appointment interval so as to decrease mean patient waiting time at the expense of greater provider idle time.

Of those doctors who had used services as a patient or patient’s relative, only 43% had used the appointment booking systems provided by their own hospital, and 89% still preferred a walk-in appointment. We believe this is because doctors may have a kind of privilege not only in their knowledge of disease, but also in the way that they can access medical resources in their own hospital compared to ordinary patients. For them, walking into the hospital where they work, even without an appointment, is not difficult. These phenomena also reduce the satisfaction of ordinary patients because of unfair preferential treatment of others. So the hospital needs to improve its services to make them fairer and better. We also found that doctors were significantly more satisfied with the service currently provided than their patients were. Doctors may think that patients always exaggerate how long they wait, and they also sometimes tended to focus on the presenting symptoms, believing that patients’ other needs were less important. However, these beliefs may have poor or limited accuracy, which is important when assessing doctors’ behavior or ability in the social–psychological realm [29]. In addition, a more active and autonomous role for the patient has recently been advocated that involves increased patient control, reduced physician dominance, and more mutual participation [30]. The patient-centered approach has become the predominant model in clinical practice today. To improve patient satisfaction and the patient-physician relationship, we believe that we need to help doctors take account of others’ opinions or feedback in treating disease, to prevent them from being too disease-centered, and ignoring patients’ other needs.

However, our study had several limitations. First, we failed to collect data on the possible reasons for non-attendance and late attendance at pre-booked appointments, partly because the majority of people who failed to attend their appointments or were late declined to give an explanation. Late and non-attendance will disrupt the orderliness of medical care and wastes limited medical resources [31]. It can further increase mean patient waiting times and so decrease patient satisfaction. Strategies that may increase patient punctuality, together with consideration of complexity of the patients’ symptoms, should be considered in a future study. Second, because our study participants were from a large tertiary hospital, and different hospitals may have a different WAS, our findings may not be generalizable to other hospitals. Different service volumes, a different hospital, or a different level of hospital could all contribute to different practices. It is also possible that recall bias is present, such that satisfaction with the overall visit to the hospital, or several hospitals, influenced the perception of time spent. We need to develop intervention strategies to further improve the usability of the WAS and increase convenience for patients. Furthermore, it needs to be mentioned that the survey was only delivered to patients who got care could really bias the sample. However, in China, most patients will choose to wait in line even the waiting was so long and bad. Few people will exit because of feeling poor or of urgent. So, we thought that the sampling bias caused by those situations must be minor to consider.

## Conclusions

In all kinds of public hospitals, particularly high-level public hospitals, reductions in waiting times for medical services could help promote patients’ satisfaction. The use of tools such as WAS can help to reduce total waiting time, and substantially increase patients’ satisfaction with outpatient services. However, the efficiency of this system has not been fully recognized by ordinary patients and doctors, and needs promotion, as very few patients actually used It. Hospitals and other health service providers should make more effort to promote and encourage the use of this effective system, such as enhancing system service quality and information variety, distributing the registration demand evenly, predicting patient throughput and waiting time with computer simulation. Strategies need to provide to help doctors to take others’ opinions or feedback into consideration when treating patients to minimize the gap between patients’ and doctors’ opinions. These findings may provide useful information for both practitioners and regulators, and improve recognition of this efficient and useful booking system, which may have far-reaching and positive implications for China’s ongoing reforms.

## Competing interests

The authors declare that they have no competing interests.

## Authors’ contributions

The six authors are justifiably credited with authorship, according to the authorship criteria, and all have approved submission of this manuscript. YH was responsible for the conception, design, analysis and interpretation of data, and drafting of the manuscript; ZM was responsible for the conception, design, acquisition of data, analysis and interpretation of data, and drafting of the manuscript; ZC was responsible for the acquisition of data, and critical revision of the manuscript; SQ and CQ were responsible for analysis and interpretation of data, and critical revision of the manuscript; and ZY was responsible for acquisition of data, and critical revision of the manuscript.

## Pre-publication history

The pre-publication history for this paper can be accessed here:

http://www.biomedcentral.com/1472-6947/14/49/prepub

## Supplementary Material

Additional file 1 Changhai outpatient experience questionnaire. Click here for file

Additional file 2 Changhai Doctor’s Experience Questionnaire. Click here for file
