# Supplementary material for: Questionnaire survey about use of an online appointment booking system in one large tertiary public hospital outpatient service center in China
Source: BMC Med Inform Decis Mak. 2014 Jun 9;14:49. doi: 10.1186/1472-6947-14-49 (PMC4059480; doi:10.1186/1472-6947-14-49)
Supplement: Additional file 1 — Changhai outpatient experience questionnaire. [file 1472-6947-14-49-S1.doc]

**CHANGHAI OUTPATIENT EXPERIENCE QUESTIONNAIRE**

**No： Date:**

Dear Sir/Madam,

To improve the service of our outpatient center, we are honored to invite you to attend our questionnaire survey about the experience in our center. Your opinions are very valuable for us. Your personal information will be kept confidential. Thank you for your support!

Best regards,

Changhai Hospital Outpatient Service Center

**PART A GENERAL INFORMATION**

**A1 YOUR CONSULTANT CLINIC**

□General clinic □Specialist Clinic

**A2 GENDER**

□Male □Female

**A3 AGE (YEARS OLD)**

□<18 □18-25 □26-35 □36-45 □46-55 □56-65 □>65

**A4 MARRIAGE**

□Single □Married □Divorced / Separated □Widowed

**A5 OCCUPATION：**

□Civil servants □Staff □Workers/service personnel

□Teacher/researcher/doctor/lawyers and other professionals

**□**farmer □private owner □Student □The emeritus and retired

□Unemployed □Others

**A6 EDUCATION LEVEL：**

□Post graduated or above □Undergraduate or Junior College

□Technical secondary school or senior □high school □Junior high school

□Primary school or below

**A7 PLACE OF RESIDENCE：**

□Local □Not local, immigrated from other cities

□Not local, just visit Shanghai

**A8 WAY OF PAYING：**

□Out-of-pocket □Medical insurance □Others payment

**A9 MONTHLY INCOME PER CAPITA (Yuan):**

□<1000 □1001-3000 □3001-5000 □5001-10000 □>10000

**A10 IS THIS YOUR FIRST TIME TO VISIT OUR HOSPITAL?**

□Yes □No

**A11 THE REASON FOR YOUR CHOOSING OUR HOSPITAL：**

**□**Great repute **□**Convenient transportation □Good service attitude

□Excellent medical skill □Advanced technology facility □Relatively cheap

□Good environment □Others：____________________

**PART B SATISFACTION ABOUT THE OUTPATIENT SERVICE**

**B1 WAITING FOR REGISTRATION:**

□Very short □Relatively short □General □Relatively long □Very long

**B2 THE MANNER OF SERVICES STAFF WHEN THEY ANSWER YOUR QUESTIONS:**

□Very patient □Relatively patient □General □Not so patient □impatient

**B3 WAITING FOR CONSULTANT:**

□Very short □Relatively short □General □Relatively long □Very long

**B4 THE LONGEST TIME YOU CAN TOLARATE WHEN WAITING FOR CONSULTANT IS MINUTES.**

- 10 minutes □20 minutes □30 minutes

□40 minutes □50 minutes □60 minutes

**B5 SATISFACTION ABOUT THE TIME OF DOCTOR’S CONSULTANT:**

□Very satisfied□Relatively satisfied □General □Not so satisfied□Unsatisfied

**B6 THE EXLPANATION OF DOCTORS ABOUT THE DIAGNOSIS AND THERAPY:**

□Very detailed□Relatively detailed □General □Not so detailed□Too simple

**B7 SATISFACTION ABOUT THE PROFESSIONAL LEVEL OF DOCTOR:**

□Very satisfied□Relatively satisfied □General □Not so satisfied□Unsatisfied

**B8 THE MANNER OF NURSE IN OUR OUTPATIEN SERVICE CENTER:**

□Very patient □Relatively patient □General □Not so patient□Impatient

**B9 THE PROFESSIONAL LEVEL OF NURSE IN OUR OUTPATIENT SERVICE CENTER:**

□Very good □Relatively good □General □Not so good□Bad

**B10 THE TIME FOR DISPENSARY TO TAKE PHARMACY:**

- Very short □Relatively short □General □Relatively long□Very long

**B11 THE SERVICE OF STAFFS IN OUTPATIENT DISPENSARY:**

□Very satisfied□Relatively satisfied □General □Not so satisfied□Unsatisfied

**B12 WAITING FOR GENERAL LABORATORY TEST:**

- Very short □Relatively short □General □Relatively long□Very long

**B13 WAITING FOR ULTRASOUND EXAM:**

- Very short □Relatively short □General □Relatively long□Very long

**B14 TIME FOR MAKING APPOINTMENT OF SPECIAL IMAGE EXAMINATION SUCH AS CT OR MRI:**

- Very short □Relatively short □General □Relatively long□Very long

**B15 WAITING FOR CHARGE:**

- Very short □Relatively short □General □Relatively long□Very long

**B16 SATISFACTION ABOUT THE RESULTS OF THERAPY:**

□Very satisfied□Relatively satisfied □General □Not so satisfied□Unsatisfied

**B17 SATISFACTION ABOUT THE MEDICAL EQUIPMENT IN OUTPATIENT SERVICE CENTER:**

□Very satisfied□Relatively satisfied □General □Not so satisfied□Unsatisfied

**B18 SATISFACTION ABOUT THE ORDER OF WAITING IN OUR OUTPATIENT SERVICE CENTER:**

□Very satisfied□Relatively satisfied □General □Not so satisfied□Unsatisfied

**B19 SATISFACTION ABOUT THE GENERAL ENVIRONMENTAL LAYOUT OF OUR OUTPATIENT SERVICE CENTER:**

□Very satisfied□Relatively satisfied □General □Not so satisfied□Unsatisfied

**B20 SATISFACTION ABOUT THE SERVICE IDENTIFICATION OF OUR OUTPATIENT SERVICE CENTER:**

□Very satisfied□Relatively satisfied □General □Not so satisfied□Unsatisfied

**B21 OUR OUTPATIENT SERVICE PROCESS IS:**

□Very convenient□Relatively convenient □General □Not so convenient □Not convenient

**B22 COMPARED WITH YOUR PAYMENT, THE MEDICAL SERVICES YOU GOT IS:**

□Very satisfied□Relatively satisfied □General □Not so satisfied□Unsatisfied

**B23 WILL YOU CHOOSE OUR HOSPITAL AGAIN WHEN YOU ARE ILL NEXT TIME?**

□Surely will□Probably will□Hard to say□Probably not □Won’t

**B24 SATISFACTION ABOUT THE SERVICES IN GENERAL YOU RECEIVED THIS TIME**

□Very satisfied□Relatively satisfied □General □Not so satisfied□Unsatisfied

**PART C SATISFACTION ABOUT APPOINTMENT-BOOKING SERVICES OF OUR OUTPATIENT SERVICE CENTER**

**C1 HAVE YOU EVER USED THE APPOINMENT-BOOKING SERVICES IN OUR HOSPITAL?**

□Have used.□Never used. □Have heard about it but haven’t used.

□Don’t know what is appointment booking.

**C2 IF YOU HAVE USED THE APPOINMENT-BOOKING SERVICES, WHICH KIND OF APPOINTMENT MODE DO YOU PREFER?**

□On-the-spot appointment (registration desk)

□On-the-spot appointment (consulting room)

□web-based appointment systems

□Self-help booking on machine in outpatient department

**C3 WHICH ONE DO YOU PREFER?**

□The mode that select precise time slots for appointments.

□The mode that select different consultant orders.

**C4 HOW LONG DO YOU THINK IS SUITABLE FOR APPOINT THE CONSULTANT NEXT TIME BY A SPECIALIST?**

□In 1 week. □In 2 weeks. □In 1 month. □In 2 months.

**C5 DO YOU THINK IT NECESSARY TO PROVIDE THE MODE OF APPOINMENT OF PRECISE TIME SLOTS IN GENERAL CLINICS?**

□Yes □No

**C6 SATISFACTION ABOUT THE APPOINMENT SERVICES OF OUR OUTPATIENT SERVICE CENTER:**

□Very satisfied□Relatively satisfied □General □Not so satisfied□Unsatisfied

**C7 DO YOU HAVE FURTHER IMPROVEMENT SUGGESTIONS ABOUT OUR APPOINMENT SERVICES?**

**_______________________________________________________________________**

_______________________________________________________________________
