# Supplementary material for: Questionnaire survey about use of an online appointment booking system in one large tertiary public hospital outpatient service center in China
Source: BMC Med Inform Decis Mak. 2014 Jun 9;14:49. doi: 10.1186/1472-6947-14-49 (PMC4059480; doi:10.1186/1472-6947-14-49)
Supplement: Additional file 2 — Changhai Doctor’s Experience Questionnaire. [file 1472-6947-14-49-S2.doc]

**CHANGHAI DOCTOR’S EXPERIENCE QUESTIONNAIRE**

**No： Date:**

Dear Sir/Madam,

To improve the service of our outpatient center, we are honored to invite you to attend our questionnaire survey about the experience in our center. Your opinions are very valuable for us. Your personal information will be kept confidential. Thank you for your support!

Best regards,

Changhai Hospital Outpatient Service Center

**PART A GENERAL INFORMATION**

**A1 PROFESSIONAL TITLE**

□Attending doctor or Resident □Associate Professor □Professor

**A2 TYPE OF YOUR CLINIC**

□General clinic □Specialist Clinic

**A3 GENDER**

□Male □Female

**A4 SPECIALTY**

□Surgery □Internal medicine

□Gynecology and obstetrics Department, Otolaryngology Department, Stomatology Department, Craniocerebral surgery Department, and Burns surgery Department

□Neurology Department and Epidemiology Department

□Imageology Department and Experimental Diagnosis Department

□Other Departments

**PART B SATISFACTION ABOUT THE OUTPATIENT SERVICE**

**B1 SATISFACTION ABOUT THE GENERAL ORDER OF WAITING IN OUR OUTPATIENT SERVICE CENTER:**

□Very satisfied□Relatively satisfied □General □Not so satisfied□Unsatisfied

**B2 SATISFACTION ABOUT THE GENERAL ENVIRONMENTAL LAYOUT OF OUR OUTPATIENT SERVICE CENTER:**

□Very satisfied□Relatively satisfied □General □Not so satisfied□Unsatisfied

**B3 SATISFACTION ABOUT THE SERVICE IDENTIFICAATION OF OUR OUTPATIENT SERVICE CENTER:**

□Very satisfied□Relatively satisfied □General □Not so satisfied□Unsatisfied

**B4 OUR OUTPATIENT SERVICE PROCESS IS:**

□Very convenient□Relatively convenient □General □Not so convenient □Not convenient

**B5 SATISFACTION ABOUT THE SERVICES PROVIDED BY OUR OUTPATIENT SERVICE CENTER**

□Very satisfied□Relatively satisfied □General □Not so satisfied□Unsatisfied

**B6 THE MOST REASONABLE TIME PROVIDED BY DOCTORS YOU THINK DURING THE CONSULTANT TO ONE PATIENT ARE _____MINUTES FOR GENERAL CLINIC AND _____MINUTES FOR SPECIALIST CLINIC.**

**B7 WILL YOU INTRODUCE YOUR FRIENDS AND RELATIVES TO OUR OUTPATIENTS SERVICE CENTER WHEN THEY NEED TO SEE A DOCTOR?**

□Definitely will.□Probably will. □Hard to say. □Probably won’t.

□Definitely won’t.

**B8 SATISFACTION ABOUT THE GENERAL MEDICAL SERVICES PROVIDED BY OUR OUTPATIENTS SERVICE CENTER TO PATIENTS**

□Very satisfied□Relatively satisfied □General □Not so satisfied□Unsatisfied

**B9 SATISFACTION ABOUT THE GENERAL ASSISTED SERVICES PROVIDED BY OUR OUTPATIENTS SERVICE CENTER TO DOCTORS**

□Very satisfied□Relatively satisfied □General □Not so satisfied□Unsatisfied

**PART C SATISFACTION ABOUT APPOINTMENT-BOOKING SERVICES OF OUR OUTPATIENT SERVICE CENTER**

**C1 DO YOU THINK IT NECESSARY TO PROVIDE VARIOUS FORMS OF APPOINMENT REGISTRATION SERVICES OF SPECIALIST CLINICS FOR PATIENTS?**

□Yes □No

**C2 DO YOU THINK IT NECESSARY TO PROVIDE APPOINMENT REGISTRATION SERVICES OF GENERAL CLINICS FOR THE SECOND-TIME-VISIT PATIENTS?**

□Yes □No

**C3 WILL YOU PROVIDE PATIENTS APPOINTMENTS DIRECTLY AT CONSULTING ROOMS?**

□Yes □No

**C4 WILL YOU PROVIDE PATIENTS APPOINMENTS OF OTHER SPECIALIST CLINICS?**

□Yes □No

**C5 HAVE YOU EVER USED THE APPOINMENT SERVICES PROVIDED BY OUR OUTPATIENS SERVICE CENTER FOR YOUR FRIENDS AND RELATIVES?**

□Have used.□Never used. □Have heard about it but haven’t used.

□Don’t know what is appointment booking.

**C6 IF YOU HAVE USED THE APPOINMENT-BOOKING SERVICES, WHICH KIND OF APPOINTMENT MODE DO YOU PREFER?**

□On-the-spot appointment (registration desk)

□On-the-spot appointment (consulting room)

□web-based appointment systems

□Self-help booking on machine in outpatient department

**C7 WHICH ONE DO YOU PREFER?**

□The mode that select precise time slots for appointments.

□The mode that select different consultant orders.

**C8 HOW LONG DO YOU THINK IS SUITABLE FOR APPOINT THE CONSULTANT NEXT TIME BY A SPECIALIST?**

□In 1 week. □In 2 weeks. □In 1 month. □In 2 months.

**C9 DO YOU THINK IT NECESSARY TO PROVIDE THE MODE OF APPOINMENT OF PRECISE TIME SLOTS IN GENERAL CLINICS?**

□Yes □No

**C10 SATISFACTION ABOUT THE APPOINMENT SERVICES OF OUR OUTPATIENT SERVICE CENTER:**

□Very satisfied□Relatively satisfied □General □Not so satisfied□Unsatisfied

**C11 DO YOU HAVE FURTHER IMPROVEMENT SUGGESTIONS ABOUT OUR APPOINMENT SERVICES?**

_______________________________________________________________________

_______________________________________________________________________
